# The role of Tre6P and SnRK1 in maize early kernel development and events leading to stress-induced kernel abortion

**DOI:** 10.1186/s12870-017-1018-2

**Published:** 2017-04-12

**Authors:** Samuel W. Bledsoe, Clémence Henry, Cara A. Griffiths, Matthew J. Paul, Regina Feil, John E. Lunn, Mark Stitt, L. Mark Lagrimini

**Affiliations:** 1 EAG Laboratories, 4780 Discovery Drive, Columbia, MO 65201 USA; 2 Hawkesbury Institute for the Environment, Western Sydney University, Locked Bag 1797, Penrith, NSW 2751 Australia; 3 Rothamsted Research, West Common, Harpenden, Hertfordshire, AL5 2JQ UK; 4 Max Planck Institut fϋr Moleckulare Pflanzenphysiologie, Potsdam (OT) Golm, Germany; 5 Department of Agronomy and Horticulture, University of Nebraska-Lincoln, 377I Plant Science, Lincoln, NE 68583-0915 USA

**Keywords:** Maize, kernel culture, drought, starvation, sugar metabolism, trehalose-6-phosphate, SnRK1

## Abstract

**Background:**

Drought stress during flowering is a major contributor to yield loss in maize. Genetic and biotechnological improvement in yield sustainability requires an understanding of the mechanisms underpinning yield loss. Sucrose starvation has been proposed as the cause for kernel abortion; however, potential targets for genetic improvement have not been identified. Field and greenhouse drought studies with maize are expensive and it can be difficult to reproduce results; therefore, an *in vitro* kernel culture method is presented as a proxy for drought stress occurring at the time of flowering in maize (3 days after pollination). This method is used to focus on the effects of drought on kernel metabolism, and the role of trehalose 6-phosphate (Tre6P) and the sucrose non-fermenting-1-related kinase (SnRK1) as potential regulators of this response.

**Results:**

A precipitous drop in Tre6P is observed during the first two hours after removing the kernels from the plant, and the resulting changes in transcript abundance are indicative of an activation of SnRK1, and an immediate shift from anabolism to catabolism. Once Tre6P levels are depleted to below 1 nmol∙g^−1^ FW in the kernel, SnRK1 remained active throughout the 96 h experiment, regardless of the presence or absence of sucrose in the medium. Recovery on sucrose enriched medium results in the restoration of sucrose synthesis and glycolysis. Biosynthetic processes including the citric acid cycle and protein and starch synthesis are inhibited by excision, and do not recover even after the re-addition of sucrose. It is also observed that excision induces the transcription of the sugar transporters SUT1 and SWEET1, the sucrose hydrolyzing enzymes CELL WALL INVERTASE 2 (INCW2) and SUCROSE SYNTHASE 1 (SUSY1), the class II TREHALOSE PHOSPHATE SYNTHASES (TPS), TREHALASE (TRE), and TREHALOSE PHOSPHATE PHOSPHATASE (*ZmTPPA.3*), previously shown to enhance drought tolerance (Nuccio et al., Nat Biotechnol (October 2014):1–13, 2015).

**Conclusions:**

The impact of kernel excision from the ear triggers a cascade of events starting with the precipitous drop in Tre6P levels. It is proposed that the removal of Tre6P suppression of SnRK1 activity results in transcription of putative SnRK1 target genes, and the metabolic transition from biosynthesis to catabolism. This highlights the importance of Tre6P in the metabolic response to starvation. We also present evidence that sugars can mediate the activation of SnRK1. The precipitous drop in Tre6P corresponds to a large increase in transcription of ZmTPPA.3, indicating that this specific enzyme may be responsible for the de-phosphorylation of Tre6P. The high levels of Tre6P in the immature embryo are likely important for preventing kernel abortion.

**Electronic supplementary material:**

The online version of this article (doi:10.1186/s12870-017-1018-2) contains supplementary material, which is available to authorized users.

## Background

Drought during flowering impacts most crops, particularly cereals such as maize, and kernel abortion resulting from osmotic stress is one of the main causes of yield loss [1–5]. Under drought, kernel abortion correlates with (i) depleted sucrose and reducing sugar levels, (ii) reduced sucrose degrading enzyme activity and transcript levels, and (iii) increased starch consumption in the kernels. Sink tissues have limited reserves of starch that can help buffer the effects of starvation; however, there is typically an insufficient amount of starch to support the ovary for an extended period of time [6]. Prolonged drought stress changes the embryo’s metabolic state, rendering it incapable of using the available sucrose [7], and the damage becomes irreversible through the activation of senescence genes which leads to programmed cell death and kernel abortion [8].

Trehalose (α-D-glucopyranosyl-(1 → 1)-α-D-glucopyranoside) and its precursor trehalose-6-phosphate (Tre6P) have been found to have many different functions including storage of chemical energy, osmoprotection, pathogen and insect defense and abiotic stress tolerance [9–13]. Trehalose is an important osmotic protectant in bacteria, fungi, and insects where it accumulates to high concentrations [14]. Most plants accumulate only trace amounts of trehalose, where it is unlikely to function as an osmoprotectant [15]. In flowering plants, trehalose is often observed in the pico to nanomolar range, suggesting trehalose and its precursor Tre6P may perform a regulatory or sensing role in plant metabolism with regards to source-sink relations [16, 17]. Recent evidence points to Tre6P having a central role in sugar sensing [18–23], leading to the proposal that Tre6P is a signal of sucrose availability, and in return regulates sucrose production and utilization [18]. It has also been proposed that Tre6P is a negative feedback regulator of sucrose levels [24, 25], perhaps through its interaction with SnRK1 [20, 26, 27]. There are numerous reports where the trehalose pathway has been engineered by constitutively overexpressing TPS or TPP [28, 29]. In some cases improved drought tolerance was observed, but no mode of action was proposed [30–32]. Perhaps the most conclusive evidence that Tre6P has a role in the plant’s response to abiotic stresses was shown using transgenic maize with ectopically produced TPP in the spikelet [33], where it was demonstrated that a reduction in Tre6P levels in the maize ear spikelet improved yield under well-watered conditions, and even more importantly under severe drought stress.

*In vitro* kernel culture has been selected as a surrogate for drought stress, providing a model system for understanding responses to water deficit and ultimately improving crop yields under drought conditions. Maize *in vitro* kernel culture was adapted to characterize the impact of sucrose starvation in immature maize kernels (as occurs during a period of water deficit stress) on growth, metabolism, energy status, gene expression and the trehalose pathway. Previous studies using *in vitro* kernel culture have examined the effects of heat stress [34], hormone synthesis [35, 36], carbon and nitrogen utilization [37], and pollination efficiency [38]. In the experiments presented here, 3 days after pollination (DAP) kernels are placed into axenic culture and subjected to 48 h of sucrose starvation followed by a 48 h recovery. Key metabolic indicators of growth and energy status including Tre6P, SnRK1, sugars, sugar phosphates, and organic acids were monitored over the course of the experiment. A detailed characterization of the expression of selected genes having a role in growth, sink strength, sucrose metabolism, sugar transport, starch metabolism provides a picture of the metabolic processes occurring in young embryos during sucrose starvation and recovery.

## Results

### Maize kernel culture

*Zea mays ssp. mays* var. B73 was grown in the field with supplemental watering as described in the Materials and Methods. Ears were hand pollinated and harvested 3 DAP. Kernels were removed by dissection, surface sterilized and plated on MS agar. Metabolites and enzymes were analyzed as described in the Materials and Methods. In developing this method, kernels were initially cultured for 15 days to assess long-term growth on sucrose, glucose, fructose and in response to sugar starvation (Additional file 1: Figure S1). Kernels on MS-only medium exhibited no significant growth; although, continued to appear healthy. Kernels cultured on sucrose, glucose or fructose more than doubled in mass over 15 d. There was no significant effect of sugar type or concentration on growth. Kernels were also cultured for 9 days on either MS salts (starvation) or MS salts supplemented with 150 mM sucrose to assess the long term impact of starvation on growth (Additional file 2: Table S1). In a separate experiment sucrose, hexoses, and starch were determined over the course of 11 days in culture, comparing MS-only medium with sucrose-enriched medium (150 mM) (Additional file 3: Figure S2). Levels of all three of these remained constant after an initial change in the first 24 h.

### Impact of starvation and recovery on metabolite levels

**Fig. 1:**
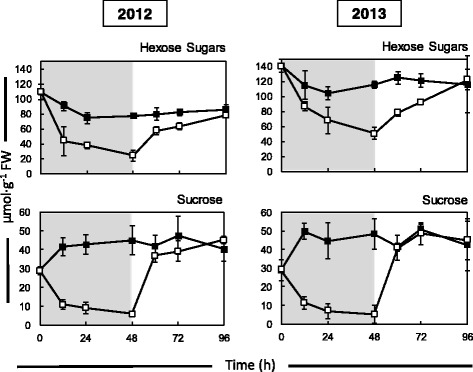
Comparison of sugar levels for kernels isolated from field grown maize (B73) from the 2012 and 2103 growing seasons. Control kernels on continuous sucrose feeding are indicated with *closed* (*black*) *squares*, and sucrose-starved kernels are indicated with *open* (*white*) *squares*. The shaded area of the graph indicates the starvation period, and the unshaded region represents the recovery stage when starved kernels were allowed to recover on sucrose-enriched medium. The initial time point (*T* = 0) is from after kernel sterilization

The impact of starvation on sugar and starch levels in the kernel was determined. Kernels harvested 3 DAP were placed on either MS-only medium or sucrose-enriched medium (150 mM) for 48 h. All kernels were then transferred to sucrose-enriched medium for another 48 h (recovery). Sucrose levels in kernels subjected to 48 h starvation were found to rapidly decrease within the first 12 h of culture, whereas sucrose levels in kernels on continuous sucrose increased initially and levelled off within the first 12 h (Fig. 1). Sucrose levels in starved kernels recovered to levels indistinguishable from kernels receiving continuous sucrose feeding. The data presented are from kernels harvested in 2012. The experiment was repeated in 2013 with no significant difference in the outcome (Fig. 1). In 2013, the calculated rates of kernel sucrose depletion and recovery were similar, 3.19 μmol∙g^−1^ h^−1^ and 3.55 μmol∙g^−1^ h^−1^ respectively. Although plants had supplemental irrigation available, the environmental conditions varied greatly between the years (Additional file 4: Table S2). The 2012 growing season was considered a drought year with the highest temperatures and growing degree days (GDD), while the 2013 growing season was more typical for the location.

### Impact of starvation on tre6p and other metabolites

**Fig. 2:**
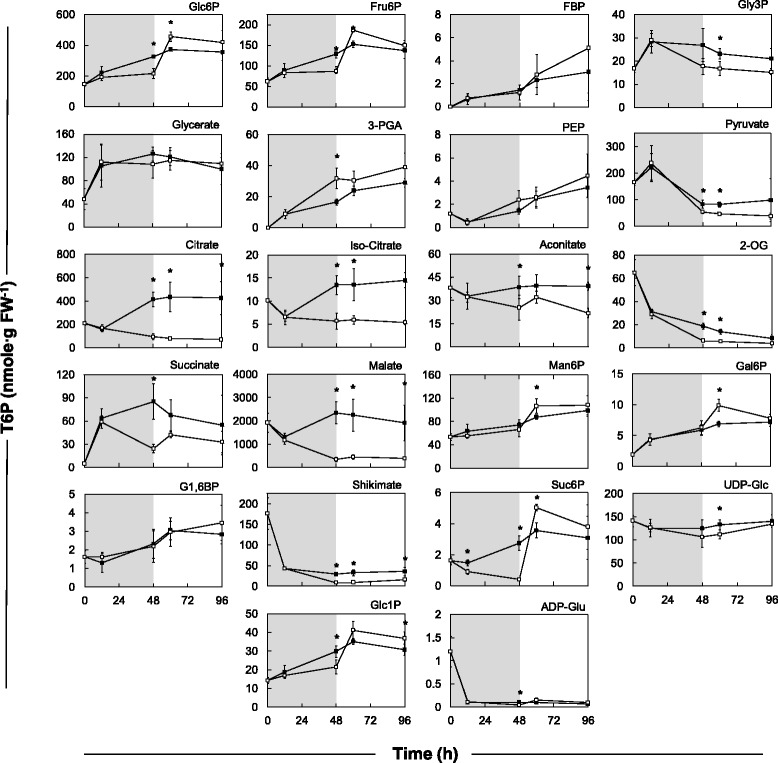
Effects of sucrose starvation on metabolite levels (on a fresh weight basis) on 3 DAP B73 kernels (2012 season). All metabolites were measured using anion exchange chromatography in tandem with mass spectrometry as described by Lunn et al. [18]. Control kernels on continuous sucrose are indicated with *closed squares*. Kernels starved for 48 h (shaded area), then allowed to recover for 48 H on 150 mM sucrose are indicated with *open squares*. Means are calculated using 4 biological replicates, consisting of 3 plants each. Significance as indicated by *asterisks* was determined by the Student’s t test (*P* < 0.05)

**Fig. 3:**
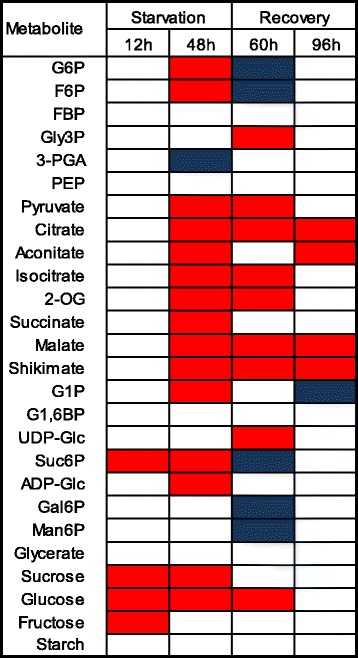
Heat map indicating the overall effect of sucrose starvation and subsequent recovery on metabolite levels for cultured maize kernels (3 DAP). ANOVA was performed on four independent biological replicates using Microsoft Excel. Significant differences, *P* < 0.05, indicated by *shaded boxes*. *Blue* indicates treatment had a positive effect over control, and *red* indicates treatment had a negative effect over control samples. Non-significant differences are indicated with *white bars*

In order to assess the effect of starvation and recovery treatment on maize kernel metabolism, an analysis of metabolites utilized in sucrose and starch synthesis, glycolysis, and the citric acid cycle was performed (Fig. 2; Additional file 5: Figure S3). Sucrose deprivation for 48 h reduced Glc and Fru to very low levels (<10 μmol∙g^−1^ FW), and this decrease was largely reversed by the addition of sucrose in the medium. The glycolytic intermediates Glc6P, Fru6P (intermediates in glycolysis, Suc synthesis and starch synthesis), fructose 1,6-bisphosphate (FBP; an intermediate in glycolysis), and glycerol 3-phosphate (Gly3P; an intermediate in the synthesis of glycerolipids) rose in the sucrose control but remained level or rose only slightly in the starvation treatment, while 3PGA and PEP rose more in the starvation treatment than in the sucrose control. Many of these differences were statistically significant at 48 h starvation. Pyruvate declined strongly after 12 h (from 150 to 60 nmol∙g^−1^ FW), with a more marked decline in the starvation treatment than the control. Many intermediates in the citric acid cycle remained high in the sucrose control but declined in the starvation treatment, including citrate, aconitate, isocitrate, 2-oxoglutarate, succinate, and malate (Fig. 3). *During the recovery from starvation, the levels of sugar phosphates in starved kernels returned to that of those maintained on continuous sucrose. 3PGA and PEP remained slightly but not significantly higher than in the sucrose control. It was striking that many organic acids did not return to initial levels after recovery on sucrose-containing medium, indicating an inhibition of pyruvate kinase and/or PEP carboxylase.

Sucrose and starch metabolism were also impacted by starvation in the detached kernels. Sucrose deprivation triggered a rapid decrease in S6P followed by a return to control levels after returning to sucrose-enriched medium, while steadily increasing in the continuously-fed kernels. The decrease in S6P during the first 8 h of starvation was more marked than for Glc6P and Fru6P which are important in both sucrose and starch synthesis (Fig. 3). There was a strong decrease in ADPGlc and shikimate in the sucrose control as well as the starvation treatment, indicating decreased aromatic amino acid synthesis, although the residual level of shikimate was slightly higher in the sucrose control. The levels of ADPGlc and shikimate remained low after the re-addition of sucrose.

**Fig. 4:**
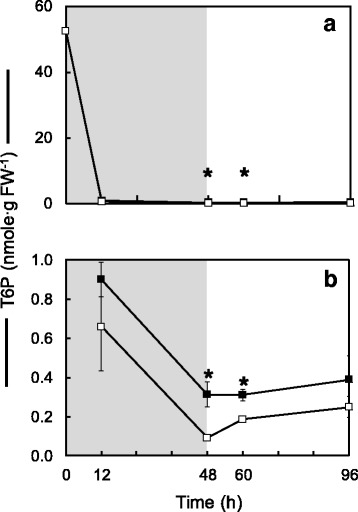
Effects of sucrose starvation on Tre6P levels in cultured maize kernels (2012 season). Tre6P content was determined for kernel tissue using anion exchange chromatography in tandem with mass spectrometry [18]. **a** Control kernels on continuous sucrose are indicated with *closed squares*. Kernels were starved for 48 h (shaded area), then allowed to recover for 48 h on 150 mM sucrose are indicated with *open squares*. **b** Region from 12-96 h is expanded for detail. Means are calculated using 4 biological replicates, consisting of 3 plants each. Significance as indicated by *asterisks* was determined by the Student’s t test (*P* < 0.05). FW, fresh weight

An exceptionally large decrease in Tre6P levels from 54 to 1 nmol∙g FW^−1^ was observed in both sucrose fed and sucrose deprived samples within the first 12 h of culture and maintained throughout the duration of the experiment (Fig. 4). *Tre6P has been shown to stimulate flux through the citric acid cycle via the activation of PEP carboxylase, thus the low levels of Tre6P in excised kernels and the lack of a recovery after re-supplying sucrose could possibly prevent the activation of this enzyme and explain the decrease in organic acids in excised kernels and the inability of sucrose to revert this decrease [25]. The zero time point was not included in Fig. 4b so that the smaller later changes in Tre6P, following the large initial decrease, could be seen more clearly. It is now possible to see that sucrose deprivation results in a small further reduction of Tre6P in kernels throughout the duration of the experiment.

### Impact of starvation and recovery on *in vitro* snrk1 activity

**Fig. 5:**
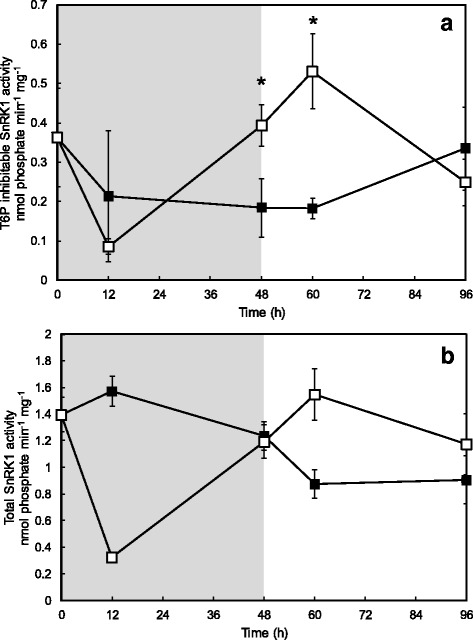
Tre6P inhibitable *in vitro* SnRK1 activity for control and sucrose-starved kernels (2012 season). SnRK1 activity was determined in tissue extracts as described by [20]. Exogenous Tre6P (1 mM) was added to determine the fraction of SnRK1 activity sensitive to Tre6P inhibition. Shaded region of graph indicates starvation period for treated kernels, and unshaded region indicates recovery period. Control samples are indicated with *filled squares* (*black*) and starved kernels are indicated with *open squares* (*white*). *Asterisks* indicate significant difference from controls (*P* < 0.05; *n* = 3). Standard error is shown with *vertical bars*

In order to assess the impact of the starvation and recovery treatments on the stress and sucrose starvation sensor SnRK1 in cultured maize kernels, *in vitro* SnRK1 activity was determined. It was previously shown that SnRK1 activity in maize kernel extracts was highly sensitive to inhibition by Tre6P [39]. Total and Tre6P-inhibitable (1 mM) SnRK1 activity was determined and graphically presented as Tre6P-inhibitable activity (Fig. 5). Control and starved kernels exhibited a drop in T6P-inhibitable SnRK1 activity after placed in culture which then returned to near initial levels by 96 h for both treatments. While this occurred monotonically for the continuously-fed control, Tre6P-inhibitable SnRK1 activity rose significantly between 12 and 48 h in the starvation treatment, and remained elevated 12 h after sucrose re-addition. Sucrose-starved kernels had significantly higher SnRK1 activity at 48 and 60 h when compared to control samples.

### Impact of starvation and recovery on gene expression

**Fig. 6:**
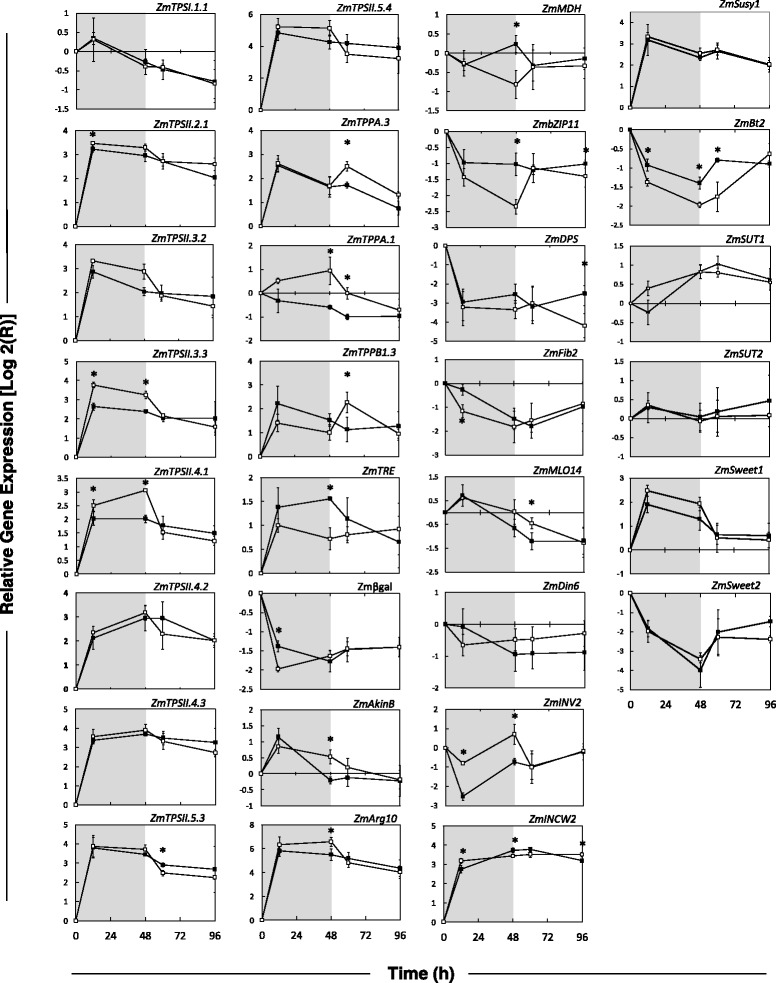
Transcript levels in *in vitro* grown B73 maize kernels subjected to sucrose starvation and recovery (2012 season). RT-qPCR was performed on total RNA extracted from kernel tissue using oligonucleotide primers specific for various genes involved in sugar metabolism, sensing and transport. *White squares* indicate kernels subjected to 48 h of sucrose starvation followed by recovery for 48 h on 150 mM sucrose. *Black squares* indicate kernels grown on continuous sucrose. The shaded area on the graph represent the period of sucrose starvation. The *white* are shows the recovery period on 150 mM sucrose. Significance as reported by the Student’s *T*-test is at α = 0.05 for 3 biological replicates, and indicated by *asterisks*. Standard error is shown with *vertical bars*

The effect of sucrose deprivation and recovery on gene expression was examined. Relative gene expression was determined by qRT-PCR for a select set of genes involved in the trehalose pathway (TPS, TPP and TRE), SnRK1 target genes and genes involved in sugar metabolism and transport (Fig. 6). Among the *TPS* genes, the ones encoding class I and Class II enzymes responded differently to sucrose deprivation. The class I gene (*ZmTPS1.1*), encoding the TPS enzyme with catalytic activity, declined gradually from 12 h onwards and did not show any significant differences in expression between continuously-fed and sucrose starved kernels. All of the class II *TPS* genes investigated were induced within the first 8 h of sucrose deprivation. There was a trend for all class II *TPS* mRNAs to rise to higher levels in the sucrose deprived samples, but only three (*ZmTPSII.2.1*, *ZmTPSII.3.3* and *ZmTPSII.4.1*) were found to have significant differences between the control and sucrose starved treatments at either the 12 h or 48 h time points (Student’s T-test, α = 0.05). This effect was reversed upon the re-addition of sucrose.

In contrast to the class II TPS genes, the *PP* genes did not respond in such a coordinated fashion. For *ZmTPPA.3*, there was a large induction of transcript levels in the first 8 h of culture. For this gene, there was no difference between control and starvation treatment within the first 48 h of starvation, but gene expression was higher during recovery for the previously starved kernels compared to controls. *ZmTPPB.1.3* gene expression was similar to that of *ZmTPPA.3*, showing a sizable induction in the first 8 h of culture (Fig. 6). Transcript levels for sucrose-deprived kernels responded to a greater extent to recovery on sucrose. *ZmTPPA.1* transcript level was not affected by culture in the presence of sucrose, but displayed a small increase as a consequence of starvation. *ZmTRE* (TREHALASE) showed an induction in the first 8 h of culture and was significantly repressed after 48 h of starvation. *ZmTRE* mRNA levels returned to that of control samples during recovery on sucrose.

The impact of the starvation and recovery treatment on the SnRK1 signalling pathway was assessed using the transcript levels of nine putative SnRK1 target genes [27]. Primers were designed for maize based on homologous sequences with Arabidopsis: 5 SnRK1 induced genes (ZmβGal, *ZmAkinβ, ZmArg10, ZmMOL14 and ZmDIN6)* and 4 SnRK1 repressed genes (*ZmMDH, ZmbZIP11, ZmDPS, and ZmFiB2*). Selected SnRK1 target genes responded to both cultivation and sucrose deprivation stress. For stress specific SnRK1 target genes, *ZmAkinβ* has been shown to be induced and *ZmMDH, ZmbZIP11* and *ZmFiB2* are repressed [27]. Among the putatively upregulated SnRK1 targets, four responded accordingly: *ZmAkinβ*, *ZmArg10, ZmDIN6* and *ZmMol14* when kernels were placed into culture. Among the putatively downregulated SnRK1 targets, five, *ZmβGal*, *ZmβZIP11*, *ZmDPS*, *ZmFib2*, *ZmMDH*, were repressed in response to culturing. However, only *ZmAkinβ*, *ZmArg10*, *ZmMDH*, and *ZmβZIP11* transcripts were significantly different in starved kernels when compared to controls at 48 h into treatment. Putative SnRK1 target gene expression responds to the addition of sucrose in the medium after 48 h of starvation in a Tre6P independent manner. This can be seen with the transcripts for *ZmAkinβ*, *ZmArg10, ZmMDH,* and most noticeably Zmβ*ZIP11*. *This Tre6P independent response to sugars is also seen with all three TPP genes (*ZmTPPA.3, ZmTPPA.1 and ZmTPPB.1.3*). After sucrose recovery (48–96 h), there was no significant difference between control and starvation treatment with the exceptions of *ZmDPS* at 96 h and *ZmMol14* at 60 h. SnRK1 target gene expression data provide further evidence that the SnRK1 pathway is responding to *in vitro* kernel starvation.

Genes critical to sucrose metabolism; vacuolar invertase (*ZmIVR2*), cell-wall invertase (*ZmINCW2*), sucrose synthase (*ZmSuSy1*), AGPase domain gene *BRITTLE2* (*ZmBT2*), and sucrose transporters (*ZmSUT1*, *ZmSUT2*, *ZmSWEET1*, and *ZmSWEET2*) were also assessed for their expression. *ZmIVR2* gene expression decreased when kernels were placed into culture, similar to the putatively downregulated SnRK1 target genes. Transcript levels; however, remained significantly higher in starved kernels than controls during the first 48 h (Fig. 6). Both *ZmINCW2* and ZmSuSy1 transcripts were induced in the first 8 h of culture for both control and treated kernels, reflecting the same pattern seen for class II *TPS* genes and putatively upregulated SnRK1 target genes. *ZmBT2* transcript decreased in the first 8 h of culture and stayed lower in sucrose-starved kernels throughout the experiment. The sucrose transporters *ZmSUT1* and *ZmSWEET1* showed higher mRNA levels for starved kernels with values approaching significance (Fig. 6). Neither *ZmSUT2* nor *ZmSWEET2* differed in expression between the control and starved kernels. The expression of three sugar transporters (*ZmSUT1*, *ZmSUT2*, *ZmSWEET1*, and *ZmSWEET2*) was followed in response to starvation stress in the immature maize kernel. The transcription of *ZmSWEET1* and *2* sugar transporters is impacted by the starvation during harvest; however, unlike the other SnRK1-regulated genes, transcription returns quickly to initial levels within 96 h. *ZmSWEET1* was strongly induced when kernels were placed into culture, yet mRNA quickly returned to initial levels upon transfer to sucrose-enriched medium. *ZmSWEET2* gene expression showed the opposite response, decreasing during starvation and increasing during recovery. The senescence indicator gene *ZmDIN6* [27] did not differ significantly between control and sucrose starved kernels.

**Fig. 7:**
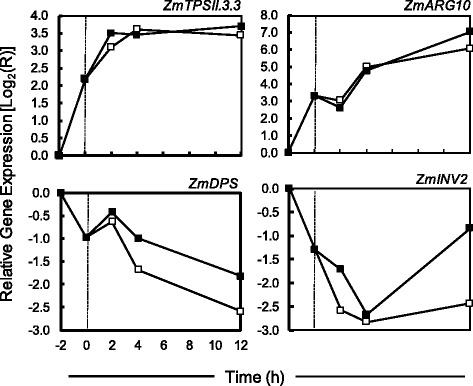
RT-qPCR was performed on total RNA extracted from maize kernels cultured *in vitro* on MS complete agar ±150 mM sucrose. *Open squares* indicate kernels starved for sucrose, and *closed squares* indicate kernels subjected to sucrose starvation. Means are plotted based on 3 biological replicates. At −2 h the ears were removed from the plant. The *vertical dashed line* indicates when kernels were placed on medium

The process of harvesting and sterilizing 3 DAP maize kernels imposes an unavoidable degree of stress. It takes 2 h from the point at which kernels were removed from the ear to plating on nutrient agar. During this 2 h window all kernels including controls were effectively starved for nutrients. It was observed that excision and sterilization impacts mRNA levels for numerous genes involved in sugar sensing, transport and metabolism. This “excision effect” is independent of the presence of sucrose in the medium. To better understand the excision effect, a separate experiment was conducted to look at gene expression in the initial hours post-excision (Fig. 7). To determine how quickly a change in transcript levels occurred after kernels were removed from the ear, we sampled at −2, 0, 2, 4 and 12 h. The −2 h time point reflects the moment the ears were removed from the plant, and the 0 h time point is when kernels are placed on nutrient medium. Quantitative RT-PCR was performed for two genes shown to be induced by excision (*ZmTPSII.3.3* and *ZmARG10*) and two excision repressed genes (*ZmDPS* and *ZmIVR2*). Transcript levels for these genes were found to change by as much as 8-fold in the two hours it took to excise, sterilize and plate the kernels (Fig. 7). Messenger RNA levels continued to increase or decrease for another 4 h before levelling off. The 3 DAP kernels were divided into three periods. Duke and Doehlert [40] looked at mRNA levels in 5 DAP maize kernels for α-*Zein*, ß-*Zein*, *Opaque-2*, *Aldolase*, *Shrunken-1*, *Waxy*, *Shrunken-2*, and *Brittle-2* at 0, 5, 10, and 15 d into culture. Transcription increased for each of these genes from 0 to 5 days, then returned to initial levels (25 °C). Although not discussed in their paper, this initial increase in transcription could be the consequence of inflicted starvation during the harvest, as observed in this paper. It is important that interpretations of the data take into consideration possible wounding or excision effects.

## Discussion

### Effect of starvation on sugar metabolism and transport

Sugar uptake is essential to sustain kernel development and leads to kernel abortion if disrupted in any way, i.e. when drought is applied around the time of pollination [41]. In the work presented here, we chose to use an *in vitro* kernel culture system (in presence or absence of sugar) as a way to mimic disruptions in sucrose supply to the kernel. We then tested how it impacted sugar and starch metabolism and sugar sensing. Sucrose levels increased and hexose levels decreased by only two thirds in the sucrose-fed control kernels. This demonstrates that in our experimental system the sucrose supplied in the media was well taken up by the kernel. It also indicates that there is a limit to how much of the sucrose and hexose can be metabolically processed, and that this metabolic processing is in equilibrium with sucrose hydrolysis. This contrasts with starved kernels in which both sucrose and hexoses decreased dramatically indicating that they are being consumed by catabolic pathways to maintain energy status. This observed equilibrium between metabolic processes and invertase activity has been shown to be maintained by hormones and hexose signals in a feedback regulation loop [42].

We observed no difference in starch levels between the control and sucrose-starved kernels over the course of the 96 h experiment. One possible explanation is that starch synthesis is irreversibly inhibited in response to the initial 2 h starvation period. The rapid decline in the expression of the AGPase gene (*ZmBt2*) as well as lower levels of the starch synthesis intermediate ADP-Glc in both the control and starved kernels supports this explanation. An alternative explanation is that embryos in early development are not in the grain filling stage, where most of the starch is synthesised [43]. Interestingly, elevated 3-PGA levels were observed. The glycolytic intermediate 3-PGA, is known to allosterically activate AGPase (regulatory step in starch synthesis) and is generally correlated with high sucrose availability resulting in starch biosynthesis [44–46]. However, in this study the opposite is observed. Lack of starch accumulation, reduced sucrose levels, repressed *ZmBt2* mRNA, and low starch biosynthesis metabolites, in particular the dedicated intermediate, ADPGlc, all indicate that starch biosynthesis is being inhibited. Although 3-PGA is high in sucrose-deprived kernels and normally would allosterically activate AGPase, AGPase is also redox-activated as it adjusts to the supply of available carbon [47], sucrose starvation is most likely the reason why the *ZmBT2* gene is turned off to a greater extent in the sucrose-deficient kernels even in the presence of elevated 3-PGA.

Whole plant studies have shown that drought stress impacts the metabolism of sucrose, and is negatively correlated with IVR2 and INCW2 enzymatic activity and mRNA transcript levels [31, 48, 49]. These enzymes, as well as Susy1, are responsible for sucrose cleavage into hexose in order for them to be uptaken by the kernels. In our experiments, we observed that 48 h of starvation induced a strong accumulation of *ZmIVR2* transcripts, a small decline of *ZmINCW2* transcripts, and did not affect *ZmSusy1* transcripts. This indicates that similarly to *ZmSWEET1* and *ZmSUT1*, *ZmIVR2* might be regulated by sucrose availability and induced upon starvation in an attempt to increase the uptake of sugar by the kernel. It also indicates that *ZmIVR2* might have a more important function than *ZmINCW2* and *ZmSusy1* in that response. This hypothesis correlates with previous findings showing that sucrose synthase (Susy) is more important in sucrose catabolism during later development, when most starch synthesis occurs, than in early development [42, 43, 50].

The SWEET transporters have been recently discovered as a new class of sugar transporters enabling passive diffusion of either sucrose or hexose through membranes according to the concentration gradient of sugars. They have therefore been postulated to be the missing transporters enabling apoplastic sucrose phloem unloading and sugar uptake by sink tissues through passive diffusion [49]. However, little is known about their specific function and localization in different plant tissues and across species [51, 52]. In contrast to the SWEET transporters, the sucrose-H^+^ symporters (SUT/SUC) enable the active transport of sucrose through membranes, driven by ATPase generated proton gradient (Carpaneto et al. 2005). SUTs are responsible for apoplastic phloem loading in source tissues and the active uptake of sugars into sink tissues including during grain filling [53]. Here we show that the transcript levels of both *ZmSWEET1* and *ZmSUT1* tend to be induced by excision and in vitro culture, but also by 12 to 48 h of sucrose starvation. This indicate that these transporters could be involved in sugar uptake by the kernels, and that they might be regulated at the transcript level by sucrose availability. The slight elevation in *ZmSWEET1* and *ZmSUT1* transcripts under starvation might be a compensatory response of the kernel in order to import more sugars and enable survival. Evidence reviewed by Ayre [54] suggests that some sucrose transporters, such as *ZmSUT1* [55], may be able to switch between functions of import and export depending on the needs of the specific plant tissue, allowing for separate functions in source and sink tissues. It has been suggested by Aoki et al. [56] that sucrose transporters may be required in non-photosynthetic organs to provide carbohydrates to growing tissues. Based on this assumption, the elevated mRNA observed in the starved kernels for *ZmSUT1* and *ZmSWEET1* may be tissue specific and important as reserves are consumed to provide for the metabolic needs of the plant experiencing sucrose starvation. Considering that this study separates the developing kernel from its physiological source tissue and that sucrose transporters are often tissue specific [56], it is not surprising that analysis on whole kernel samples will produce conflicting results as it measures all the components simultaneously.

### Role of Tre6P and the trehalose genes in the starvation response

In context of the trehalose biosynthetic pathway, the results generally agree with what is known about this pathway. Tre6P levels have been shown to be highly correlated with sucrose (and Suc6P) concentration [18, 24, 57–60], and further shown to inhibit SnRK1 activity in developing tissues [20, 26, 27]. Yadav et al. [24] and Lunn et al. [18] proposed that, under a given set of conditions, the ratio of Tre6P to sucrose stays constant in that one controls the other in a Tre6P/sucrose nexus that regulates key metabolic processes important in the production and utilisation of sucrose and growth. Tre6P was shown to follow sucrose levels closer than any of the other sugars. They also proposed that that the sensitivity of the sucrose-Tre6P relationship is constant for a particular tissue, developmental stage and environmental condition, but can and does change if any of these three factors changes such as imposed stress [25, 57]. In the data presented here the correlation between sucrose and Tre6P did not always hold true. This likely resulted from a change in the condition or stage of the tissue between time zero and the 12 h time point. A similar interaction between sucrose and Tre6P levels has been observed before for shading stress in maize seedlings [61], and for salt stress in mature maize plants [39]. Based on Zhang et al. [20] who found that *in vitro* SnRK1 activity is inhibited by Tre6P at the concentrations found in kernels, it can be proposed that this large decrease of Tre6P will activate SnRK1 in both the control and starved kernels, presumably during the excision and preparation period (2 h). *The starved kernels later have a higher level of Tre6P-inhibitable SnRK1 activity, measured in vitro, indicating an additional mechanism may also promote SnRK1 activity in starved excised kernels. A similar interaction between sucrose and Tre6P levels has been observed before for shading stress in maize seedlings [61], and for salt stress in mature maize plants [39]. These findings add to the increasing evidence of the diverse functions of Tre6P and bring to light more of how little is known about this disaccharide and the need for further exploration of the trehalose pathway.

The results of this study agree mostly with the expected trends for the putative SnRK1 target genes. *ZmβGal* was an exception, being repressed initially; however, by 48 h there was no difference between the control and starved kernels. The correlation of Tre6P to SnRK1 target gene expression suggests that Tre6P inhibits SnRK1 activity. As the level of Tre6P drops during sucrose starvation there is a positive correlation with putative SnRK1 downregulated genes and a negative correlation with upregulated genes (Additional file 6: Table S3). The negative correlation between *TPS* class II genes and Tre6P is further evidence that Tre6P is involved in SnRK1 regulation as it is known that class II *TPS* genes are also targets of SnRK1 [27].

The responses of metabolites also support the activation of SnRK1 by starvation, and the involvement of the trehalose pathway in carbohydrate metabolism of kernels in the early stages of development. The reduction in sucrose synthesis, starch and citric acid cycle intermediates indicates a switch in metabolism consistent with an activation of SnRK1 as reviewed in [62–64]. The intermediary metabolite shikimate is an indicator of growth via its role in aromatic amino acid synthesis. During sucrose starvation shikimate levels are depleted. Low shikimate can be attributed as an indicator of growth arrest via the inhibition of key metabolic regulatory proteins (14–3-3 proteins) [65]. Glycolytic intermediates will be formed during sugar metabolism and also during catabolism, and their level will depend upon the relationship between the rate of formation and utilization. Interestingly, glycolytic intermediates were high in sucrose-fed kernels and in starved kernels, remained relatively high and rose further sucrose was re-supplied. This contrasts with organic acids and shikimate which remain low, indicating that the metabolic condition of the starved kernel continues in a state of growth arrest for the duration of the experiment. Perhaps if given more time for recovery the metabolites would return to control values. An alternative explanation is that the kernels have started down an irreversible path leading to abortion [8, 66] and will not recover.

The only intermediate that showed significantly increased levels during starvation was 3-PGA. 3-PGA levels were previously shown to decrease in maize leaves upon starvation induced by extended shading [57, 61]. When Arabidopsis seedlings were subjected to sucrose starvation and then fed sucrose in a recovery phase the seedlings experienced a decrease in 3-PGA during the first 3 days of recovery before levels increased [66]. Their study also showed rapid increases in sucrose, hexoses, sugar phosphates, and ATP. This observation is consistent with the recovery phase for kernels (Fig. 3). 3-PGA is important in both glycolysis and the Calvin cycle; however, we can presume that most of the 3-PGA in the immature kernel comes from glycolysis. In glycolysis, 3-PGA is an important intermediate in the oxidation of hexose sugars to pyruvate to be used in either respiration or biosynthesis [67]. The build-up in PEP suggests that the consumption of this metabolite by pyruvate kinase and PEP carboxylase is restricted. Low PEP carboxylase activity could be due to the low Tre6P levels in excised kernels, given that high Tre6P leads to activation of this enzyme [68]. Via the reversible enolase and phosphoglycerate mutase reactions, accumulation of PEP would also be expected to lead to a similar accumulation of 3-PGA, accounting for the parallel increases in PEP and 3-PGA in excised kernels.

The build-up in 3-PGA may indicate the location of the restriction in glycolysis could be a result of lower phosphoglycerate mutase activity from repressed carbon availability. However, a more probable explanation is that the elevated 3-PGA concentration can be attributed to changes in glycerate availability from other sources in the plant tissue. The catabolic conversion of serine derived from the mitochondria and peroxisome may be the source of glycerate that would result in higher 3-PGA concentrations in sucrose deprived kernels [68].

### Role of the *TPS* gene family in the stress response

In plants, the concentration of trehalose is too low to be useful as an osmoprotectant; however, the trehalose pathway has been shown to be essential in plants especially with regards to the activity of the *TPS* class I gene. The class I TPS (TPS1) has been confirmed to have enzymatic activity through complementation in yeast mutants deficient for TPS [69–71], and knocking out the *AtTPS1* gene in Arabidopsis demonstrates its vital importance in embryo development and vegetative growth, and that the numerous class II TPSs are unable to compensate for the absence of TPS1 [72, 73]. In our experiments, the transcription of maize *ZmTPSI.1.1* in 3 DAP kernels was found to be unresponsive to sucrose starvation. This is consistent with our previous findings that *ZmTPSI.1.1* was unresponsive to salt stress in 3 DAP kernels [39]. A similar observation was made in Arabidopsis root and leaf tissue where *AtTPS1* gene expression was found to be unresponsive to a wide range of abiotic stresses including drought, salt, cold, wounding, and heat [74]. This indicates that although it is essential to trehalose metabolism, TPS1 gene expression is not regulated at the transcript level. Given that the *TPS1* gene is transcribed constitutively in most tissues, the catalytically active TPS is likely regulated via post-translational modification or binding of regulatory ligands [75, 76].

To date, the function of the class II TPS proteins remains unclear. As stated previously, they fail to reproducibly complement yeast TPS1 mutations, and they have thus far not been demonstrated to have *in vitro* enzymatic activity [71]. Regardless of the uncertainty of their function, class II TPSs show strict developmental and tissue specific gene regulation, strong diurnal regulation, and sensitivity to a variety of abiotic stressors [39, 74, 77]. The maize *ZmTPSII.3.3* gene was shown to associate with the kernel expansion trait in popcorn [78]. The class II TPS proteins 5, 6 and 7 from Arabidopsis were found to be phosphoproteins and bind to 14–3-3 proteins when phosphorylated at Ser22 and Thr49 [76]. Unlike *ZmTPSI.1.1*, all of the maize class II *TPS* genes we followed were induced at the transcript level within the first 8 h of kernel culture, regardless of treatment. Class II *TPS* genes behaved similarly to the stress-inducible putative SnRK1 responsive genes with respect to the initial 2 h starvation, suggesting that SnRK1 has a role in the regulation of the class II genes in maize, as previously described in Arabidopsis [64].

### Tre6P Attenuates the Stress Response

**Fig. 8:**
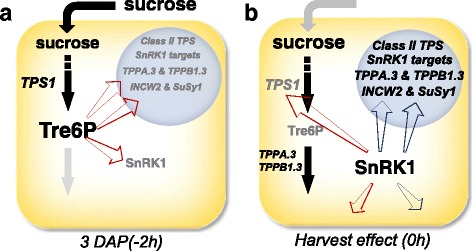
This cartoon summarizes the events occurring during the two hours required to harvest, surface sterilize, and plate 3 DAP kernels on medium. In (**a**), kernels are on the plant being fed on sucrose obtained from photosynthesis. Cellular sucrose (20 μmol∙g^−1^ FW) and Tre6P levels are high (54 nmol∙g^−1^ FW) and SnRK1 activity is low, as is the transcription of putative SnRK1 target genes, class II TPS genes, TPP genes, INCW2 and SuSy1. Two hours after ears are removed from the plant (**b**) Tre6P levels drop below 1 nmol∙g^−1^ FW, SnRK1 activity is high, and the transcription increases for putative SnRK1 target genes, class II TPS genes, TPP genes, INCW2 and SuSy1

A two-hour period of starvation occurs for all samples between removal of the ear from the plant, though excising and surface sterilization of kernels, until plating on medium. Although tissue carbohydrate reserves are not completely depleted during this two hour imposed starvation stress, it is sufficient stimulus to see a significant impact on gene expression, particularly of putative SnRK1 target genes (Fig. 8). This effect on transcript levels is maintained throughout the experiment (96 h) for most genes regardless of whether or not sucrose is provided in the medium, likely because the Tre6P that typically attenuates the activity of SnRK1 and the expression of starvation responsive genes drops from a base level in the 3 DAP kernel of 54 nmol∙g FW^−1^ to <1 nmol∙g FW^−1^. Although there was a significant change in SnRK1 target gene expression in less than 12 h after kernels were excised from the cob, there was no significant change in total or Tre6P-inhibitable *in vitro* SnRK1 activity for control kernels. The activation of SnRK1 activity after kernel excision is likely a consequence of the precipitous drop in Tre6P levels, and not due to changes in SnRK1 protein levels. Actually total *in vitro* SnRK1 activity drops significantly after 12 h of starvation, even as SnRK1 is continuing to influence *in vivo* gene expression; although, *in vitro* Tre6P-inhibitable SnRK1 activity in sucrose starved kernels is significantly higher than in control kernels (>2-fold). In many ways the imposed starvation stress in maize kernels parallels that which is seen in the maturing wheat kernel [58]. Tre6P levels drop precipitously, SnRK1 becomes de-repressed, putative SnRK1 target genes are expressed, and significant changes in *TPP* and class II *TPS* gene expression are observed.

It is likely that the drop in Tre6P levels observed in starved maize kernels occurs because of the activation of one of the numerous trehalose phosphate phosphatases. All ten of the *TPP*s from Arabidopsis have been confirmed to have enzymatic activity through complementation of a yeast mutant [17]. Also, a chloroplast-associated redox-sensitive TPP from Arabidopsis (AtTPPD) was found to positively influence stress tolerance [79]. Salt had no effect on *TPP* gene expression in 3 DAP maize kernels [39]. The maize *TPP RAMOSA 3* gene (*ZmTPPB.2.1*) is suspected to have a role in apical meristem growth and inflorescence development [80]. *RAMOSA 3* is involved in branching in maize, and when *RAMOSA 3* is knocked out of the maize genome unusual branching is often the result [81]. Each of the *TPP* genes responded differently to starvation stress, possibly indicating these genes have different functions in specific cell types found in a tissue sample [61]. It was observed; however, that starvation results in a large induction in the transcription of the *ZmTPPA.3* gene. Recently, a rice ortholog of *ZmTPPA.3* (*OsTPP1*) was ectopically over-expressed in maize pedicel tissue [33]. This resulted in a drop in ear spikelet (5 days before pollination) Tre6P levels from 48 to 18 nmol∙g FW^−1^, an increase in sucrose levels from 35 to 50 μmol∙g^−1^ FW, and improvement in drought tolerance. Interestingly, the protein coding sequence for *ZmTPPA.3* was used to predict its subcellular localization (Wolf PSORT, Plant-mPLoc, and CropPlan), which suggested a high probability that the enzyme is localized to the chloroplast (data not shown). Tre6P is predominantly in the cytosol and a small amount associated with the chloroplast for Arabidopsis leaves grown in the light [82]. This suggests that Tre6P perhaps moves between subcellular compartments. This is consistent with a system of Tre6P transporters, similar to Glc6P transporters in *E. coli* [83, 84] and human [85]. It is currently not known if TPP is post-translationally activated.

## Conclusions

The impact of 48 h of starvation on 3 DAP maize kernels remains after two days’ recovery on sucrose-enriched medium. In the field this is equivalent to a rainfall being unable to reverse drought-induced kernel abortion. Future work will examine genetic variability amongst maize inbreds with respect to the capacity of kernels to recover from starvation.

Kernel excision triggers a 50-fold drop in kernel Tre6P, which does not recover even after 96 h on sucrose-enriched medium. The removal of Tre6P suppression of SnRK1 activity results in the transcription of several putative SnRK1 target genes, and analyses of metabolite levels reveals a metabolic transition from biosynthesis to catabolism. This observation demonstrates the sensitivity of the maize inbred B73 to drought-induced kernel abortion, and highlights the importance of Tre6P in the metabolic response to starvation. We can speculate that the extremely high levels of Tre6P seen in the immature seed are needed to suppress SnRK1 activity, and further that the route to kernel abortion may begin with the precipitous drop in Tre6P and the resulting activation of SnRK1.

The drop in Tre6P corresponds to a large increase in transcription of *ZmTPPA.3*, indicating that this specific enzyme may be responsible for the de-phosphorylation of Tre6P, and thus a potential target for genetic and biotechnological improvement of drought tolerance in maize and other cereals.

## Methods

### In vitro maize kernel culture

This method was modified from the procedures of [34, 35, 37, 86] (Additional file 7: Figure S4). Metabolic and gene expression studies were performed in order to look at the impact of environmental conditions on kernel metabolism, verify that kernels continue to grow and develop normally in culture and determine the usefulness of this method as a surrogate for drought stress. In two sequential years the maize inbred B73 was grown in replicated plots with equal spacing and surrounded by a border to account for equal competition and shading. The maize inbred B73 was grown in replicated field plots in 2012 and 2013, watered twice a week to maintain available water content in the soil above 50%. The growing seasons of 2012 and 2013 were noticeably different (Additional file 4: Table S2). The 2012 growing season was considered a drought year which saw the highest temperatures and growing degree days (GDD) compared for all three years. May and July in 2012 had the hottest days with average high temperatures 15 and 11% respectively above the mean, and GDDs 28 and 17% respectively above the 20-year mean. In addition, rainfall was the lowest for 2012 with precipitation 54% lower than the mean. The 2013 growing season was milder with temperatures consistent within 4% and the GDD within 7% from the mean. Precipitation for 2013 was 16% below the mean. Each plot was replicated in three plantings, each a week apart to insure that enough pollen could be obtained. The plants were watered twice a week via sprinkler to minimize stress and ensure that available water content in the soil was kept above a 50% threshold. Emerging shoots were covered by shoot bags to ensure no pollination can take place, and ears were pollinated by hand with B73 pollen. Three days after pollination, kernels were harvested from each of the 4 randomized rows from the most centrally located plants to ensure equal competition of available ground nutrients as well as shading effects. Ears were harvested, kept cool and hydrated in a beaker filled with water until preparation was complete (<2 h). Kernels were prepared by hand excision of the center one-third of the ear, and removal of kernels and a small portion of cob tissue. Sufficient cob tissue (cob to kernel ratio of 6:1) was required to permit nutrients to be taken up by the immature kernel [86]. The immature kernels were sterilized and grown in sucrose rich (150 mM sucrose, 1% MS agar), or sucrose deficient media (1% MS agar) on square petri dishes with equal spacing to ensure that there was no unequal competition between the developing kernels for the duration of the experiment [34, 35, 87]. The embryos were kept at 24 °C in the dark for 48 h, then transferred to plates with sucrose for another 48 h. Replicate samples are removed every 8–12 h, weighed and immediately frozen in liquid N_2_.

### Carbohydrate and metabolite analysis

In order to evaluate the effect of sucrose starvation, excised kernels were cultured on MS medium without a carbon source for 48 h then allowed to recover on sucrose-enriched medium for another 48 h. Kernels cultured for 96 h on continuous sucrose served as a control treatment. Frozen tissues (20–100 mg) were ground to a fine powder (30–60s) with a Tissue Lyser II (Qiagen). Sucrose, fructose, and glucose were extracted using lactose as an internal standard [18]. Starch was extracted from the pellet generated during the extraction of soluble sugars, and hydrolyzed with amylase to produce glucose. All samples were analyzed by high-pressure capillary ion chromatograph system (ICS-5000, PA-20 column; ThermoFisher Dionex) using a 1 μl injection volume and 45 mM KOH eluent. Sugar peaks were identified in comparison with known sugars, and data were analyzed using the formula previously described with L/G ratio of 1/100 for kernel and cob [61]. T6P and other phosphorylated metabolites were quantified by anion-exchange liquid chromatography linked to tandem mass spectrometry [68].

### In vitro SnRK1 activity

Kernel tissue (200 mg) was ground in a mortar and pestle under liquid N_2_. Soluble proteins were extracted in 600 μL of ice-cold homogenization buffer (100 mM Tricine-NaOH, pH 8.0, 25 mM NaF, 5 mM dithiothreitol, 2 mM tetrasodium pyrophosphate, 0.5 mM EDTA, 0.5 mM EGTA, 1 mM benzamidine, 1 mM phenylmethylsulfonyl fluoride, 1 mM protease inhibitor cocktail (Sigma P9599), phosphatase inhibitors (PhosStop; Roche) and insoluble polyvinylpyrrolidone to 2% (*w*/*v*). The homogenate was centrifuged (13,000 g at 4 °C), and the supernatant (250 μL) was desalted on Illustra NAP-5 columns (GE Healthcare) pre-equilibrated with homogenization buffer. SnRK1 activity was determined as described [20]. Duplicate samples received Tre6P to a final concentration of 1 mM to determine the percentage of in vitro SnRK1 activity inhibited by Tre6P.

### Messenger RNA analysis by qRT-PCR

RNA isolation from maize tissue and qRT-PCR was performed as described in [61]. RT quality and absence of genomic DNA contamination was then checked by semi quantitative PCR on 5 μL of cDNA (1/100 dilution) in a final volume of 25 μL, using ZmEF1–1 alpha primers (For: AGA CTC ACA TCA ACA TTG TGG TCA T, Rev.: GTT GTC ACC TTC AAA ACC AGA GAT T) designed around an intronic region and GoTaq® DNA Polymerase (Promega, USA) as recommended by the supplier. For each time point and biological replicate, qRT-PCR reaction was repeated 3 times. Experiments were performed on 3 biological replicates. Three out of 8 reference genes [39] were selected for normalization of gene expression using the Genorm software [88]. Relative gene expression was calculated using the method of [89]. Primer efficiency was determined using the method described by Pfaffl et al. [90].

## Additional files

Additional file 1: Figure S1. Kernels harvested 3 DAP were assessed for growth on various concentrations of sucrose, glucose and fructose during long term culture (15 d). After sterilization, kernels were plated on MS agar and incubated in the dark at 24 °*C. medium* contained either only MS salts, or MS salts supplemented with 100, 200 or 300 mM sucrose, glucose or fructose as a carbon source. Individual kernels were weighed at day zero of culture and at day 15. (XLSX 225 kb)

Additional file 2: Table S1. Kernels harvested 3 DAP were assessed for the effect of long term (9 d) sucrose starvation on kernel growth (g^−1^ fw). After sterilization, B73 kernels were plated on MS agar and incubated in the dark at 24 °C. Control kernels on continuous sucrose are indicated by SUC (150 mM), and sucrose starved kernels are indicated by NS (no sucrose). Individual kernels from three seperate ears were weighed each day and returned to the medium. (XLSX 11 kb)

Additional file 3: Figure S2. Kernels harvested 3 DAP were tested for long term viability in culture. After sterilization, kernels were plated on MS agar and incubated in the dark at 24 °C. Control kernels on continuous sucrose (150 mM) are indicated with closed (black) squares and sucrose starved kernels are indicated by white (open) squares. Means are plotted using 4 biological replicates consisting of 3 plants each. (XLSX 24 kb)

Additional file 4: Table S2. Growing conditions for field grown maize plants. Monthly and cumulative temperature (°C), growing degree days (GDD) (base 10 °C for *Zea mays*), and precipitation (cm) for the University of Nebraska-Lincoln campus field plots for the 2012, 2013, and 2014 growing seasons. The mean is determined on the 20-year average. (XLSX 15 kb)

Additional file 5: Figure S3. Effect of sucrose starvation on *in vitro* cultured kernels (3 DAP). Kernels were excised from field grown maize plants 3 days after pollination. After sterilization, kernels were plated on MS agar and incubated in the dark at 24 °C. Control kernels on continuous sucrose (150 mM) are indicated with closed (black) squares and sucrose starved kernels are indicated by white (open) squares. The shaded area highlights the 48 h period of starvation, and the unshaded area indicates the period on recovery on sucrose. Means are calculated using 4 biological replicates consisting of 3 plants each, and significance as reported by the Student’s *T*-test is at α = 0.05. (XLSX 85 kb)

Additional file 6: Table S3. Heat map of correlation coefficients between Tre6P levels and gene expression. Coefficients of correlation were determined for control kernels continuously fed sucrose, and kernels starved for 48 h using a Pearson comparison test (*n*-4). Positive correlations are indicated by shades of blue and negative correlations are indicated by shades of red. (XLSX 11 kb)

Additional file 7: Figure S4. Maize kernel culture. Kernels were prepared by hand excision of the center one-third of 3 DAP ears (A), and removal of kernels and a small portion of cob tissue (B), maintaining sufficient cob tissue to permit nutrients to be taken up by the immature kernel. Immature kernels were sterilized and grown in sucrose rich (150 mM Sucrose, 1% MS agar), or sucrose deficient media (1% MS agar) on petri dishes with equal spacing to ensure that there was equal competition for nutrients (C). Embryos were kept at 24 °C in the dark for 48 h, then transferred to plates with sucrose for another 48 h. Replicate samples are removed every 8–12 h, kernels were removed from cob tissue (D & E), weighed, and immediately frozen in liquid N_2_. (XLSX 310 kb)

## Abbreviations

3-PGA: 3-phosphoglyceraldehyde
ADP-Glc: Adenine diphosphate glucoside
ASI: Anthesis silking interval
DAA: Days after anthesis
DAP: Days after pollination
FBP: Fructose bisphosphate
Fru: Fructose
Fru6P: Fructose-6-phosphate
GDD: Growing degree days
Glc: Glucose
Glc1P: Glucose-1-phosphate
Glc6P: Glucose-6-phosphate
Gly3P: Glyceraldehyde-3-phosphate
HPIC: High pressure ion chromatography
HPIC: High pressure ion chromatography
SnRK1: Sucrose non-fermenting-1-Related protein Kinase 1
TPP: Trehalose phosphate phosphatase
TPS: Trehalose phosphate synthase
TRE: Trehalase
Tre6P: Trehalose-6-phosphate
UDPG: Uridine diphosphate glucose
